# Antidiabetic activity of the ethyl acetate fraction of *Ficus lutea* (Moraceae) leaf extract: comparison of an *in vitro* assay with an *in vivo* obese mouse model

**DOI:** 10.1186/s12906-016-1087-z

**Published:** 2016-03-31

**Authors:** Oyinlola O. Olaokun, Lyndy J. McGaw, Ilse Janse van Rensburg, Jacobus N. Eloff, Vinny Naidoo

**Affiliations:** Department of Paraclinical Sciences, Phytomedicine Programme, University of Pretoria, Private Bag X04, Onderstepoort, 0110 South Africa; Biomedical Research Centre, Faculty of Veterinary Sciences, University of Pretoria, Private Bag X04, Onderstepoort, 0110 South Africa

**Keywords:** Glucose uptake, Insulin secretion, Digestive enzyme inhibition, Diet induced obesity, Diabetes, Weight control

## Abstract

**Background:**

*Ficus lutea* crude acetone leaf extracts were previously shown to stimulate glucose uptake and insulin secretion of established cells and, inhibit α-amylase and α-glucosidase activities.

**Methods:**

For this study, *F. lutea* acetone extracts were subjected to solvent-solvent fractionation to yield fractions with differing polarities (hexane, chloroform, dichloromethane, ethyl acetate, *n*-butanol and water) in an attempt to obtain a more potent fraction with *in vitro* and probably *in vivo* activity.

**Results:**

Among these fractions, the ethyl acetate fraction had the highest total polyphenol content (100.5 ± 1.6 mg GAE/g dried extract) and α-glucosidase inhibitory activity (126.8 ± 30.6 μg/ml). It also stimulated the highest glucose uptake of C2C12 muscle cells and decreased extracellular glucose concentration of H-4-II-E liver cells with low cytotoxic activity. The ethyl acetate fraction (10.88 ± 0.55 μg/L at 250 μg/ml) enhanced insulin secretion in RIN-m5F pancreatic β-cells to the same degree as the positive control glibenclamide (11.09 ± 0.07 μg/L at 1μM). While fractionation increased α-glucosidase inhibition and glucose uptake of cells, in the ethyl acetate fraction, the α-amylase inhibition and insulin secretion decreased. The weight reducing and glucose control potential of the ethyl acetate fraction in an obese mouse model, important factors in the amelioration of type II diabetes was determined. The extract had no statistical significant weight reducing activity.

**Conclusion:**

A major finding was the decrease in the area under the curve of the glucose concentration over time in animals that were treated with both a change in diet and with the plant extract. This is linked to increased glucose uptake within the cells, the most likely mechanism is either an increased insulin response or increased insulin secretion.

## Background

Diabetes mellitus is a disease characterised by chronic hyperglycaemia due to defects in insulin secretion, insulin action or both [1]. Of the different forms of diabetes, based on pathophysiology, type II diabetes is the predominant form of the disease accounting for 90 % of all cases globally in both developed and developing countries. The pathogenesis of type II diabetes is complex and involves an interaction between genetic susceptibility and environmental factors, especially the consumption of diets that promote obesity coupled with physical inactivity. With an increase in sedentary lifestyles globally, type II diabetes mellitus is considered one of the most rapidly growing non-communicable diseases and is currently the fourth to fifth leading cause of death [2]. The World Health Organisation (WHO) has estimated that 171 million people were diagnosed with diabetes mellitus in 2000 and predicts an increased prevalence of 366 million cases by 2030 if no action is taken [3].

While many therapies such as lifestyle intervention with moderate exercise and weight loss with pharmacologic agents can control many aspects of type II diabetes, none has so far convincingly demonstrated an ability to decrease the progressive loss of pancreatic insulin secretory function that eventually requires exogenous insulin supplementation [4, 5], or the development of other pathological complications. Other concerns associated with the use of conventional treatments are their inherent side effects such as abdominal discomfort, anorexia, diarrhoea, hepatic and renal impairment [4, 5]. As a result, newer and more effective drugs need to be developed. One proposed method to find a safe and effective therapeutic agent would be to use a medicinal plant which has a history of being safe, effective, low cost and having a lower incidence of adverse effects, although the latter may just be an unproven perception [6].

Numerous phytochemicals have been recognised for their potential health benefits. Recent studies have indicated that the consumption of polyphenol-rich remedies are associated with reduced risks for a variety of non-communicable diseases including diabetes [7]. In a previous study, we could show that a *Ficus lutea* acetone extract contains polyphenol compounds that influenced the inhibition of α-amylase and α-glucosidase activities [8], as well as glucose uptake of cells and insulin secretion [9]. For this study we evaluated *F. lutea* acetone extracts by fractionation to determine if potentizing is possible both *in vitro* assays and *in vivo* in an obese murine model.

## Methods

### Extraction and fractionation

The leaves of *F. lutea* Vahl, an attractive tree with wide glossy leaves were collected at the Manie van der Schijff Botanical Garden (University of Pretoria), South Africa in February 2009, and a voucher specimen (PRU 074568) was conserved in the HGWJ Schweikerdt Herbarium of the University of Pretoria. The dried ground leaves of *F. lutea* (5 g) were extracted and dried as previously reported [9, 10]. Thereafter, the weighed dried crude acetone extract was re-dissolved in 50 % acetone in water, and successively and exhaustively partitioned (by liquid-liquid extraction) with hexane, chloroform, dichloromethane, ethyl acetate, *n*-butanol and water (in order of increasing polarity). Each fraction was extracted thrice and concentrated using a rotary evaporator (Büchi R-114). The partially dried fractions were dispensed into pre-weighed glass vials and allowed to dry at room temperature under a stream of cold air. The dried fractions were stored at 4 °C until they are used. The ethyl acetate fraction was prepared every two weeks for animal experiments to reduce oxidation.

#### *In vitro* assays

##### Total polyphenol content

The total polyphenol content was determined as previously described [8]. Briefly, to 100 μl of fraction (1 mg/ml in 80 % methanol) was added 500 μl Folin-Ciocalteu reagent (1/10 dilution) and 1000 μl of distilled water. The mixture was allowed to stand for 1 min at room temperature, where after 1500 μl of 20 % Na_2_CO_3_ solution was added. The final mixture was shaken and incubated for 1 h in the dark at room temperature. The absorbance was measured at 760 nm using a plate reader (Versamax Molecular Devices). Gallic acid was used as standard. All assays were done in triplicate on one day and repeated on three different occasions. The results are expressed as mg of gallic acid equivalent (GAE) per gram dry weight of crude extract.

##### α-Amylase inhibition assay

The α-amylase inhibition assay made use of the method described by Ali et al. [11] as earlier reported [8]. To 40 μl of each fraction (10 mg/ml in DMSO) were added 200 μl of ice cold porcine pancreatic α-amylase (type VI) at 4 U/ml and 160 μl of distilled water in a screw-up plastic tube. The content was gently mixed and incubated at 25 °C for 5 min. This was followed by the addition of 400 μl of potato starch (0.5 % w/v) in 20 mM phosphate buffer (pH 6.9), and incubation for 3 min. An aliquot of the mixture (200 μl) was dispensed into a separate tube containing 100 μl of DNS colour reagent solution (96 mM 3, 5-dinitrosalicylic acid, 5.31 M sodium potassium tartrate in 2 M NaOH) and was placed into an 85 °C water bath. After 15 min, the tube was removed from the water bath, cooled and the content was diluted with 900 μl distilled water. α-Amylase activity was determined by measuring the absorbance of the mixture at 540 nm. Acarbose was used as positive control, for solvent control (100 % enzyme activity), fraction was replaced by DMSO while for the blank enzyme solution was replaced with distilled water and the same procedure was carried out as above. The assays were run in triplicate and repeated thrice. The α-amylase inhibition activity was expressed as:

$$ \begin{array}{l}\%\kern0.5em \mathrm{Inhibition}\kern0.5em =\kern0.5em 100\kern0.5em \times \kern0.5em \left(\frac{\Delta {\mathrm{A}}_{\mathrm{Control}}\kern0.5em \hbox{-} \Delta {\mathrm{A}}_{\mathrm{Sample}}}{\Delta {\mathrm{A}}_{\mathrm{Control}}}\right)\\ {}\Delta {\mathrm{A}}_{\mathrm{Control}}\kern0.5em =\kern0.5em {\mathrm{A}}_{\mathrm{Test}}\kern0.5em \hbox{-} \kern0.5em {\mathrm{A}}_{\mathrm{Blank}}\\ {}\Delta {\mathrm{A}}_{\mathrm{Sample}}=\kern0.5em {\mathrm{A}}_{\mathrm{Test}}\kern0.5em \hbox{-} \kern0.5em {\mathrm{A}}_{\mathrm{Blank}}\end{array} $$

##### α- Glucosidase inhibition assay

The α-glucosidase inhibition assay was done by using the method of Bhandari et al. [12] as earlier described [8]. Sucrose (200 μl of a 56 mM solution) dissolved in 0.1 M phosphate buffer (pH 7) was mixed with 100 μl of fraction (2.5 mg/ml in 50 % DMSO) in a test tube. After pre-incubation at 37 °C for 5 min, 200 μl of rat intestinal α-glucosidase solution was added. Acarbose was used as positive control while solvent (50 % DMSO) replaced the fraction to represent 100 % enzyme activity. After thoroughly mixing, the samples were incubated for 20 min and then the reaction was stopped by adding 750 μl of 2 M Tris-HCl buffer (pH 6.9). The amount of liberated glucose was determined by glucose oxidase method and absorbance was measured at 540 nm. All assays were done in triplicate and repeated thrice. The α-glucosidase inhibitory activity was calculated as done for α-amylase activity above.

##### Calculation of EC_50_

The concentration of fractions of *F. lutea* that inhibited 50 % of the activity of α- amylase and α-glucosidase termed half maximal effective concentration (EC_50_) was determined by the Kinetica 5 (Thermo) programme as earlier reported [8].

##### Glucose uptake

The level of glucose uptake was determined using the methods of Yin et al. [13] and Deutschlander et al. [14] as earlier described [9]. C2C12 muscle myocytes (25 000 cells/ml) and H-4-11-E hepatoma cells (30 000 cells/ml) suspended in DMEM supplemented with 0.25 % BSA were seeded (200 μl) into wells of 96-well plates. After incubation at 37°C in a 5 % CO_2_ incubator for 4 days (C2C12) and 2 days (H-4-11-E), the cells in the plates were used for the glucose uptake assay. Prior to the glucose uptake assay, the fractions were dissolved in DMSO to a concentration of 100 mg/ml which was further diluted (15 μl – 250 μl) with appropriate growth medium before use in the assay. The cells were incubated for 1 h (C2C12) and 3 h (H-4-11-E) with the various treatments. Insulin (Lantus) (1, 10 μU) served as the positive control. After the incubation period, the glucose concentration in the medium was determined by the glucose oxidase method with absorbance measured at 540 nm. The assays were run in triplicate and repeated thrice. The percentage glucose uptake was calculated as the percentage change in absorbance in comparison to the untreated cells using the following formula:

$$ \begin{array}{l}\%\kern0.5em \mathrm{Glucose}\kern0.5em \mathrm{uptake}\kern0.5em =\kern0.5em 100\kern0.5em \times \kern0.5em \left(\frac{\Delta {\mathrm{A}}_{\mathrm{Control}}\kern0.5em \hbox{-} \Delta {\mathrm{A}}_{\mathrm{Sample}}}{\Delta {\mathrm{A}}_{\mathrm{Control}}}\right)\\ {}\Delta {\mathrm{A}}_{\mathrm{Control}}\kern0.5em =\kern0.5em \mathrm{Absorbanc}{\mathrm{e}}_{\left(\mathrm{Untreated}\kern0.5em \mathrm{cells}\right)}\\ {}\Delta {\mathrm{A}}_{\mathrm{Sample}}=\kern0.5em \mathrm{Absorbanc}{\mathrm{e}}_{\left(\mathrm{Treated}\kern0.5em \mathrm{cells}\right)}\end{array} $$

The toxicity of the fractions to the C2C12 muscle and H-4-II-E liver cells was concurrently determined after the exposure using the 3-(4,5-dimethylthiazol-2-yl)-2,5-diphenyl tetrazolium bromide (MTT) assay [15]. In short, after the removal of the supernatant from cells for glucose assay, the cells were rinsed once with phosphate buffered saline (PBS) (200 μl). Then fresh medium (200 μl) containing 30 μl of MTT (5 mg/ml in PBS) was added to each well and further incubated for 4h. Subsequently, the medium was carefully aspirated without disturbing the formazan crystals and replaced with 50 μl of DMSO. The absorbance of the coloured formazan was measured at 570 nm after gentle shaking using a plate reader (VERSAmax). The percentage cell viability was calculated as the absorbance of the treated well divided by the absorbance of the solvent control well. The LC_50_ (concentration that was lethal to 50 % of the cells) were calculated from a plot of the log of concentration versus cell viability.

##### Insulin secretion assays

The amount of insulin secreted by RIN-m5F cells was determined by the method of Persaud et al. [16]. The RIN-m5F cells (100 000 cells/ml) were suspended in fresh growth medium (RPMI-1640 supplemented with 10 % FBS, 10 mM HEPES and 1 mM sodium pyruvate) and seeded (200 μl) into 96-well plates. Cells were incubated at 37 °C in a 5 % CO_2_ incubator to allow for adherence for 48 h. Thereafter, the medium was removed and cells were incubated in a glucose-free Krebs-Ringer buffer pH 7.4 supplemented with 1 mg/ml BSA and 10 mM HEPES for 2 h. The medium was removed and replaced with 100 μl of glucose-free Krebs-Ringer buffer containing ethyl acetate fraction at 4 concentrations (62.5, 125, 250 and 500 μg/ml). Glibenclamide (1, 10, 100 μM) was used as positive control. The cells were incubated for 1 h. The insulin content of supernatants was determined using rat insulin ELISA kit (DRG). The assays were run in duplicate and repeated three times. Insulin content was calculated from a calibration curve of the absorbance values for calibrators against insulin concentration using the linear equation based on the curve. The amount of insulin secreted was expressed as μg/L.

##### *In vivo* assay

Healthy 5 weeks old male CD1 mice (*n* = 40) were housed singly (to limit fighting) at the University of Pretoria Biomedical Research Centre (UPBRC) in conventional Eurostandard type II cages (Techniplast) on cloth bedding at the temperature of 22 °C (±2 °C), under controlled relative humidity (50 % – 60 %) in a light/dark cycle of 12 hours. Environmental enrichments were provided to keep the mice busy. The mice had free access to water and standard rodent chow for one week during the acclimatisation period. The animal experiment was approved by the Animal Use and Care Committee of the University of Pretoria with approval number 0V60 and in compliance with the South African National Standard on the housing and care of laboratory animals.

After acclimatisation, all mice were fed a purpose-made high caloric diet of standard rodent diet containing 42 % (w/w) fat and 36 % (w/w) sugar for about 12 weeks to induce obesity. Once obesity was attained (animals being 5 g heavier than the normal mass for the species at the specific age), mice were randomly assigned into one of the four treatment groups (*n* = 10) and kept on the diet of a particular treatment plan for the next 7 weeks. The study was undertaken as two independent experiments as follows; Treatment Plan 1: High caloric diet (HCD) intake including the ethyl acetate fraction at 1 mg/kg (w/w) to determine if weight loss was possible in the presence of a high-caloric diet. Treatment Plan 2: Normal diet (ND) intake including ethyl acetate fraction at 1 mg/kg (w/w), to determine if weight loss was possible in obese animals switched to a normal caloric diet. Diets were prepared fresh every two days and animal feed was changed at regular intervals (every Monday, Wednesday and Friday) to limit oxidative change of the feed. Both treatments were compared to equivalent caloric non-medicated food

Ante-mortem parameters monitored included determining body mass, food intake and faecal mass for each mouse. For the latter the faecal material was carefully removed from the cloth bedding and weighed. The difference between feed intake and faecal weights before treatment and after the treatment period was used to estimate that specific point’s degree of nutrient absorption. Blood glucose was monitored using the glucose tolerance test (GTT). Following a 6 hour fast period, mice were treated with 2 g of glucose/kg body weight (SABAX 50 % dextrose) intraperitoneally (i.p). Blood glucose levels were measured using the Contour TS glucose test strips (Bayer) and glucometer at 5, 10, 15, 30, 60 and 90 minutes. The entire process was repeated after 6 weeks of treatment. The values of area under the glucose-time curve (AUC) were calculated with SPSS 20 (IBM). Post-mortem parameters monitored included serum chemistry parameters for alkaline phosphatase activity (ALP), alanine aminotransferase (ALT), urea, creatinine, total protein, cholesterol, globulin, albumin and triglycerides using an automatic analyser and full necropsies.

##### Statistical analyses

Statistical analyses were evaluated by one-way analysis of variance (ANOVA) and considered to be significantly different at *p*˂0.05. Non-normal data were log-transformed prior to statistical testing. When significance was found, location of significance was determined by Bonferroni and Tukey HSD multiple comparison *post hoc* tests. All analyses were undertaken in SPSS 20 (IBM). Data are presented as the mean ± standard error of mean (S.E.M.). For the glucose tolerance test differences before and after treatment were ascertained using a paired *t*-test. For the degree of absorption, differences before and after treatment were determined using a paired *t*-test, following natural logarithmic transformation of the data to obtain normality.

## Result

### *In vitro* assays

The total polyphenol content of the six fractions varied widely (Table 1), from 10.32 ± 0.82 to 100.51 ± 1.60 mg GAE/g dry weight of extract. The polyphenol content of the intermediate polarity ethyl acetate fraction was the highest (100.51 ± 1.60 mg/g dry weight of extract) and was significantly higher than the other fractions of lower or higher polarity (*p*˂0.001). This was followed by the *n*-butanol fraction (79.58 ± 0.50 mg/g dry weight of extract).

**Table 1:** The total polyphenol content and EC_50_ of α-amylase and α-glucosidase activity of fractions of *F. lutea* extract

Fractions	^a^Total polyphenol (mg GAE/g dry weight extract)	^a^α-Amylase inhibition (EC_50_) μg/ml	^a^α-Glucosidase inhibition (EC_50_) μg/ml
Hexane	14.86 ± 1.43^b^	˃1000	˃1000
Chloroform	10.32 ± 0.82^b^	˃1000	˃1000
Dichloromethane	11.83 ± 2.32^b^	˃1000	854.51 ± 56.92^c^
Ethyl acetate	100.51 ± 1.60^c^	39.53 ± 7.10^c^	126.78 ± 30.62^e^
*n*-Butanol	79.58 ± 0.50^d^	26.50 ± 1.22^d^	195.17 ± 63.60^d^
Water	13.34 ± 0.85^b^	˃1000	558.40 ± 51.67^c^
^f^Acarbose	-	7.71 ± 0.42^b^	6.28 ± 0.03^b^

^a^Values are means of triplicate determinations done three times (*n* = 9) ± standard error;

^b,c,d,e^No significant difference between extracts with same value, but significant difference *p* ˂ 0.05 between different values

^f^Positive control for α-amylase and α-glucosidase

The concentration of fractions that inhibited 50 % of α-amylase and α-glucosidase activities (EC_50_) is presented in Table 1. The *n*-butanol fraction was the most potent inhibitor of the α-amylase enzyme (EC_50_ = 26.50 ± 1.22 μg/ml) followed by the ethyl acetate fraction (39.53 ± 7.10 μg/ml) with a significant difference between the two (*p*˂0.05). Conversely the ethyl acetate fraction was the most potent inhibitor of the α-glucosidase enzyme (EC_50_ = 126.78 ± 30.62 μg/ml) followed by the *n*-butanol fraction (195.17 ± 63.60 μg/ml) with a significant difference between the two (*p*˂0.05*)*. The EC50 values for the non-polar extracts in both assays were higher than the highest concentration tested. The EC_50_ for acarbose against activities of α-amylase and α-glucosidase were much lower at 7.71 ± 0.42 μg/ml and 6.28 ± 0.03 μg/ml respectively.

**Fig. 1:**
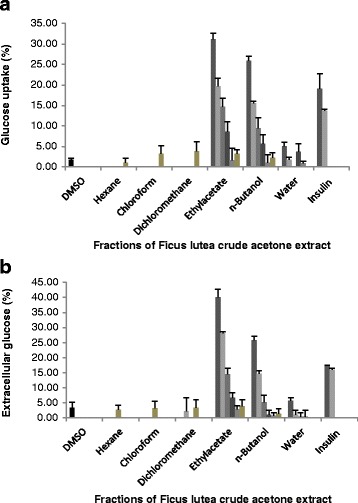
Glucose concentration (**a**) C2C12 muscle cells glucose uptake and (**b**) H-4-11-E liver cells extracellular glucose (expressed as percentage of untreated control cells ± standard error of mean, *n* = 9) exposed to the fractions of *F. lutea* extract and insulin as positive control (100 μU = 3.64 μg/ml). Fractions:  500,  250,  125,  63,  31,  15 μg/ml; Insulin:  10, 1μU

The effect of the fractions (15 μg/ml – 500 μg/ml) on glucose uptake in C2C12 muscle cells is presented in Fig. 1a. Of all the fractions, only the ethyl acetate and *n*-butanol fractions significantly (*p*˂0.001) increased glucose uptake in the cells in a dose related manner with the ethyl acetate fraction inducing the highest glucose uptake of 31.2 ± 1.5 % at 500 μg/ml followed by the *n*-butanol fraction at 25.9 ± 1.2 % as the same concentration. The maximum uptake stimulated by the ethyl acetate and *n*-butanol fractions at 500 μg/ml was significantly (*p˂*0.05) higher than the 19.1 ± 3.7 % stimulated by insulin (positive control) at 10 μU. The effect of fractions (15 μg/ml – 500 μg/ml) on extracellular glucose concentration of H-4-11-E liver cells is presented in Fig. 1b. Only the ethyl acetate and n-butanol fractions significantly (*p*˂0.001) decreased extracellular glucose concentration in a dose related manner with the ethyl acetate fraction leading to the greatest decrease (40.0 ± 2.8 %) at 500 μg/ml, followed by the n-butanol fraction (25.9 ± 1.4 %) at the same concentration.

The cytotoxic effect of the fractions against C2C12 muscle and H-4-II-E cells is presented in Table 2. Among all the fractions, the hexane fraction is the least cytotoxic to C2C12 muscle cells (the LD50 is greater than 1000 μg/ml) while the chloroform (564.2 ± 15.7 μg/ml) and ethyl acetate (562.9 ± 4.9 μg/ml) fractions are the least cytotoxic to the H-4-II-E liver cells. However, the *n*-butanol fraction is the most cytotoxic fraction to both cells; the C2C12 muscle (53.3 ± 6.2 μg/ml) and H-4-II-E liver (148.9 ± 2.5 μg/ml) cells.

**Table 2:** Cytotoxicity activity of fractions of *F. lutea* extract

Fraction	C2C12 muscle cells (LD_50_) μg/ml	H-4-II-E liver cells (LD_50_) μg/ml
Hexane	>1000	406.9 ± 5.4
chloroform	236 ± 11.7	564.2 ± 15.7
Dichloromethane	478.6 ± 12.8	389.4 ± 15.6
Ethyl acetate	508.0 ± 10.3	562.9 ± 4.9
*n*-Butanol	53.3 ± 6.2	148.9 ± 2.5
Water	NT	NT

Values are means of triplicate determinations done three times (*n* = 9) ± standard error

ND: Not tested

**Fig. 2:**
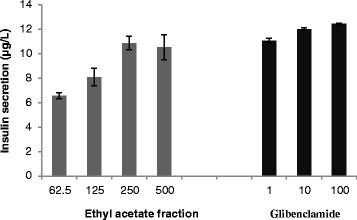
Insulin secretion of RIN-m5F pancreatic cells (expressed as percentage of untreated control cells ± standard error of mean, *n* = 6) exposed to the ethyl acetate fraction and glibenclamide as positive control (100 μM = 49.4 μg/ml) in glucose free medium

With the ethyl acetate fraction being the most active fraction in stimulating glucose uptake in the treated C2C12 muscle and H-4-II-E cells and relatively low cytotoxicity, only this fraction (62.5 μg/ml – 500 μg/ml) was further investigated for its insulin secretion activity. The RIN-m5F pancreatic cells exposed to ethyl acetate produced a dose related increase in insulin secretion (Fig. 2). The insulin release increased significantly (*p*˂0.001) from 6.58 ± 0.24 μg/L (at fraction concentration of 62.5 μg/ml) to 10.88 ± 0.55 μg/L (at fraction concentration of 250 μg/ml). The 500 μg/ml dose had no superior response to the 250 μg/ml dose, indicating that the maximum effect has plateaued. Similarly the RIN-m5F pancreatic β-cells exposed to glibenclamide (positive control) significantly (*p*˂0.001) increased insulin secretion in a dose dependent manner with the highest insulin secretion (12.44 ± 0.07 μg/L) at the concentration of 100 μM.

### *In vivo* assays

**Fig. 3:**
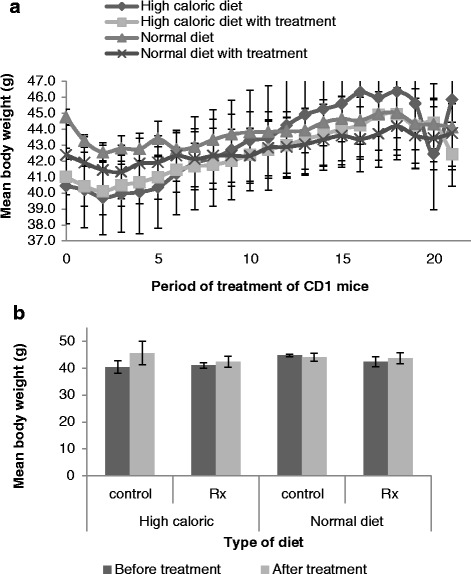
The effect of diets on body weight of CD1 mice. **a** The initial body weight at period 0 was when obese state was attained by mice prior to commencement of treatment for about 7 weeks during which body weight was measured thrice weekly until period 21 of final weight and (**b**) Body weight of mice before and after treatment on high caloric and normal diet with and with treatment (ethyl acetate fraction) (mean ± SEM)

The initial mean weight of the CD1 mice at period 0 (Fig. 3a) before commencement of treatment was 40.45 ± 2.35 g (HCD), 41.02 ± 1.12 g (HCD with treatment), 44.77 ± 0.48 g (ND) and 42.39 ± 1.88 g (ND with treatment). The mice were hereafter fed their respective diet for about 7 weeks with body weight measured three times a week when food was changed. The body weight of the control mice fed the HCD and the ND gradually increased throughout the study more than that of their comparative treatment groups, with the HCD having the greater increase. Conversely, the body weight of mice on treatment in conjunction with a HCD and ND with the ethyl acetate fraction showed a gradual decrease in body weight throughout the study, with the mice on ND and treatment having the greatest reduction. None of these differences was significant (*p* = 0.18) between the treatments and their controls. The final mean weight of mice at the end of the treatment plan was 44.14 ± 4.67 g (HCD group), 40.88 ± 0.92 g (HCD with treatment group), 45.90 ± 2.10 g (ND group) and 39.54 ± 1.34 (ND with treatment group). A comparison of the period 0 weight (initial mean weight) to the period 21 weight (final mean weight) of mice (Fig. 3b) showed that HCD mice had a significant (*p* = 0.04) increase in body weight (13.39 %), in comparison to HCD with treatment and ND with treatment (3.43 % and 3.23 % respectively). No change was seen in the animals switched to the ND.

**Fig. 4:**
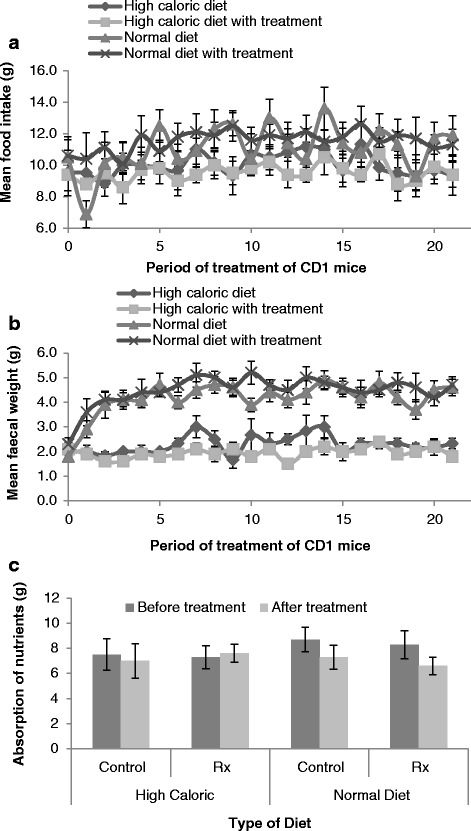
The effect of diets on food intake and feacal weight of CD1 mice. **a** Food intake and **b** Faecal weight at period 0 was when obese state was attained by mice prior to commencement of treatment for about 7 weeks during which food intake and faecal weight were measured thrice weekly until period 21 for final food intake, and (**c**) Nutrient absorption (mean ± S.E.M.) before and after the treatment period. The nutrient absorption was estimated as the difference between actual food intake and faecal weight

After induction of obesity the mean food intake (period 0) before commencement of treatment varied (9.50 ± 1.42 g, HCD group; 9.40 ± 1.01 g, HCD with treatment group; 10.50 ± 1.12 g, ND group and 10.60 ± 1.20 g, ND with treatment group) but did not differ significantly. The mice were fed their respective diets for about 7 weeks while food intake was measured three times a week when food was changed (Fig. 4a). In general the mice placed on ND ate more food than those on high caloric diet but in no case were the differences in food uptake significant. The final mean food intake at the end of the study (period 21) was 10.60 ± 1.07 g (HCD group), 9.00 ± 0.98 g (HCD with treatment group), 14.00 ± 0.53 g (ND group) and 12.20 ± 0.98 g (ND with treatment group). The initial mean faecal weight (period 0) prior to commencement of treatment varied from 2.00 ± 0.36 g (HCD group), 2.10 ± 0.28 g (high caloric with treatment group), 1.80 ± 0.20 g (ND group) to 2.30 ± 0.26 g (ND with treatment group). Mice fed on a ND had significantly higher mean faecal weights (*p*˂0.01) than those on HCD irrespective of treatment (Fig. 4b). At the end of the study the final mean faecal weight (period 21) for the mice were 2.80 ± 1.14 g (HCD group), 1.67 ± 0.33 g (HCD with treatment group), 6.25 ± 0.30 g (ND group) and 4.60 ± 0.28 (ND with treatment group). The estimated nutrient absorption per group was compared before and after treatment (Fig. 4c). No differences in nutrient absorption were present for the animals on the HCD. For the animals switched to the ND, a significant decrease in nutrient absorption was evident for both groups. For the diet change alone this was 19.3 % (*p* = 0.04) and for the diet change with treatment this was 25.8 % (*p* = 0.0097). No difference was present between the two groups switched to the normal caloric diet.

**Fig. 5:**
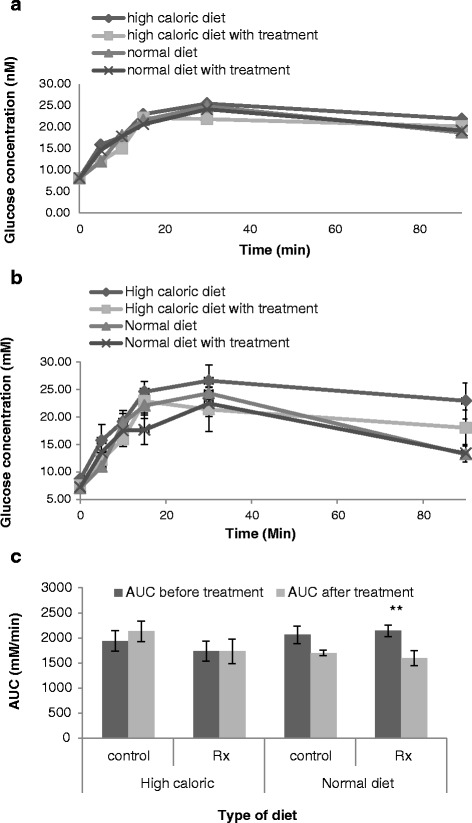
The effect of diets on blood glucose concentrations of CD1 mice. **a** Fasting blood glucose and GTT at period 0 when obese state was attained by mice prior to commencement of treatment, (**b**) Fasting blood glucose and GTT of mice after 6 weeks of treatment and (**c**) Area under the curve (AUC) of glucose concentration. AUC was calculated from curves of blood glucose levels (mM) verses time intervals (min). AUC before treatment was glucose response at period 0 when obese state was induced in mice. AUC after treatment was glucose response at period 21 after treatment (Rx). Values are the mean ± SEM. **Statistical difference between AUC before and AUC after treatment (*p˂*0.01)

The average fasting blood glucose concentration was 8.43 ± 1.16; 8.83 ± 0.57; 8.90 ± 0.88 and 8.82 ± 0.58 mM for obese mice prior to commencement of treatment placed in HCD; HCD with treatment; ND; and ND with treatment groups respectively. These values did not differ significantly (*p*˂0.05) (Fig. 5a). After 6 weeks of treatment the average fasting blood glucose concentrations for the CD1 mice did not differ significantly between the treatment groups (Fig. 5b). There was also no significant difference in the blood glucose concentration for the mice in the treatment groups after the administration of the glucose bolus. The pre-treatment and post-treatment area under the curve (AUC) of glucose concentration versus time profiles were compared (Fig. 5c), the mice in the high-caloric group showed an increase in total plasma glucose concentrations (9.97 %); the high caloric group on treatment remained unchanged; while both the normal caloric groups had a decrease (17.7 % for diet change only and 25.68 % for diet change with treatment). The difference for the high caloric groups was non-significant, for the ND change alone the difference tended towards significance (*p* = 0.07) while the change for the animals switched to the HCD with treatment was significant (*p* = 0.018).

The result of the serum chemistry parameters is presented in Table 3. All the values were within the normal range with the exception of creatinine, urea, alkaline phosphatase activity (ALP) and alanine aminotransferase (ALT). The creatinine and urea values for all the mice are higher than the normal reference values. The normal reference value is 0.63 ± 0.08 mmol/l for creatinine and 0.9 – 1.2 mmol/l for urea. In addition the ALP value for mice on HCD are higher than the normal reference range and significantly higher (*p*˂0.05) than for the other animals (128.7 ± 30.51 U/l) while the high ALT value for mice on HCD with treatment (150.2 ± 122.62 U/l) are within the normal reference interval for the CD1 mice.

**Table 3:** The effect of diets (with or without ethyl acetate fraction) on serum chemistry parameters of CD1 mice

Serum chemistry parameters	Treated Group				^a^Untreated group
	High Caloric	High Caloric with Treatment	Normal diet	Normal diet with Treatment	Standard rodent diet
Total protein (g/l)	51.3 ± 1.52	52.1 ± 0.90	52.2 ± 0.92	50.1 ± 0.94	46 – 58
Albumin (g/l)	25.2 ± 1.85	23.6 ± 1.56	24.6 ± 1.97	23.4 ± 1.06	24 – 32
Globulin (g/l)	26.2 ± 1.25	28.7 ± 1.00	27.9 ± 1.55	26.7 ± 1.02	20 – 28
Albumin/globulin ratio	1.0 ± 0.0	0.9 ± 0.11	0.9 ± 0.10	1.0 ± 0.0	0.96 – 1.36
ALT (U/l)	21.2 ± 1.33	150.2 ± 122.62*	79.0 ± 30.50	76.6 ± 28.10	66 – 170
ALP (U/l)	128.7 ± 30.51*	57.7 ± 14.44	54.5 ± 4.83	52.3 ± 3.57	72 – 118
Cholesterol (mmol/l)	4.7 ± 0.56	4.8 ± 0.36	3.9 ± 0.23	3.7 ± 0.24	3.7 – 5.7
Urea (mmol/l)	4.5 ± 0.34	4.1 ± 0.42	9.3 ± 0.50	8.4 ± 0.36	0.9 – 1.2
Creatinine (mmol/l)	9.0 ± 0.0	10.1 ± 1.11	9.0 ± 0.0	8.7 ± 0.27	0.63 ± 0.08
Triglycerides (mmol/l)	1.3 ± 0.33	1.0 ± 0.17	1.3 ± 0.21	1.7 ± 0.20	10.7 ± 0.08

*Statistical difference between treatment and control groups (*p*˂0.05)

^a^River [38]

The liver, kidneys, pancreas, heart and blood vessel to the legs were collected for histological examination. No pathological changes were observed in the kidneys, heart and blood vessel to the legs. The most consistent morphological changes in the animals were the hypertrophic changes in the endocrine islet cells of Langerhans within the pancreas and metabolic-induce vacuolation and swelling of the hepatocytes in the liver (hepatosis). Excessive fat depots were also observed in several of the animals and excessive fat is one of the predisposing factors for diabetes. These morphological changes were induced by the high caloric diet. There was no demarcation apparent between the pathological changes in mice and the diet consumed.

## Discussion

Our results revealed that the *in vitro* hypoglycaemic activities of *F. lutea* extract were in the ethyl acetate and *n*-butanol fractions (intermediate polarity) with the ethyl acetate fraction having a superior antidiabetic activity. The ethyl acetate fraction containing many different compounds at a concentration of 250 μg/ml had a similar level of activity as the positive control glibenclamide, a pure compound at a concentration of c. 50 μg/ml. This may indicate that the ethyl acetate fractions could contain compounds as effective as or better than glibenclamide.

The ethyl acetate fraction was a polyphenol-rich extract containing compounds including epiafzelechin [9], and catechins and epicatechins (unpublished data), that potently inhibited α-glucosidase activity and influenced glucose uptake of C2C12 muscle cells and H-4-II-E liver cells as well as enhanced insulin release of RIN-m5F pancreatic cell. In some *Ficus* species the polyphenol rich extracts were identified in the intermediate polar fractions either in the ethyl acetate fraction or the butanol fraction [17, 18], supporting our findings. Polyphenol-rich extracts are known to influence carbohydrate metabolism and glucose homeostasis through various mechanisms of which the most common are delaying of glucose absorption through inhibition of the activities of α-amylase and α-glucosidase thereby blunting post-prandial hyperglycaemia, stimulating of insulin release and increasing the numbers of glucose transporters [9, 19, 20].

Based on its superior *in vitro* antidiabetic activity, the ethyl acetate fraction was investigated further for *in vivo* activity. In this study obesity and its concomitant pre-diabetic condition, characterised by mild pancreatic changes and associated changes in GTT were induced in normal male CD1 mice placed on HCD *ad libitum* for a total period of 13 weeks. Hereafter they were placed in one of the four treatment plans. Treatment plan 1 was to simulate the practice of failing to reduce caloric intake in a weight loss programme while treatment plan 2 was essentially to simulate the standard practice of decreasing caloric intake in a weight loss programme.

One of the problems with induction diabetic studies is proving that the animals are pre-diabetic for proper evaluation of the efficacy results. For this study we believe that the animals were pre-diabetic for the following reasons. The mice on the high caloric induction diet showed an increase in body mass and obesity, with elevated blood glucose concentrations. The trend towards diabetic nephropathy which develops as a result of chronic hyperglycaemia was also present in the study animals as seen with the increase in plasma creatinine and urea concentrations [21, 22]. The histopathology report also indicated that the animals were pre-diabetic as the animals had moderate to excessive fat deposit in the abdominal cavity, mild to moderate cell swelling with vacuolated changes within the cytoplasm of the hepatocytes with accumulation of fatty acids suggesting metabolic-induced fatty acid changes of the liver and enlargement of the pancreatic islets of β-cell [23, 24].

With insulin resistance being fundamental in the pathogenesis of type II diabetes, intervention is initially aimed towards improvement in tissue sensitivity/responsiveness with modulation of weight being suggested as a better treatment modality. Weight loss is known to improve insulin sensitivity and overall glycaemic control, and to decrease mortality rates [25]. While a change in diet alone can result in moderate weight loss, this is difficult to achieve in the long term as weight lost is slowly regained due to the physiological abnormalities induced by obesity [25]. As a result it has been suggested that weight loss may be enhanced through combination with pharmacological agents that can reduce or control weight by altering appetite, metabolism, decreased fat absorption or consumption of calories [26, 27]. Of these, much emphasis has been place on the inhibition of the glucosidase and amylase enzymes. Inhibition of these enzymes, particularly of α-glucosidase in the small intestine, which catabolises non-absorbable complex carbohydrates into absorbable monosaccharides will modulate blood glucose concentration and weight gain [28]. The α-glucosidase and α-amylase enzyme inhibitors regulate blood glucose, especially postprandial blood glucose by limiting the rate of starch and sucrose metabolism, delaying the absorption of glucose and fructose in the gastrointestinal tract and the gastric emptying rate, which further alters the secretion of insulin [28]. In addition, the limiting the rate of starch metabolism may promote weight loss by reducing the digestive availability of carbohydrate derived calories [29].

The search for newer weight controlling agents from plant polyphenols may be an alternate source or adjunct means of controlling diabetes [30]. For this reason the obese mice were exposed to the ethyl acetate fraction of *F. lutea*, in an attempt to establish the effect of the extract in the pre-diabetic condition in the presence or absence of dietary modification. Since the animals were allowed *ad lib*. access to food, the test extract was included within the food. The reason for this was that acarbose, a known α-glucosidase inhibitor, is recommended for use on consumption of the main meals [31]. For this study, the mice placed on treatment without a change in their caloric intake maintained a steady weight throughout the study in comparison to further increases in the control animals which received the HCD alone. This suggests that in cases of high caloric intake, the ethyl acetate fraction of *F. lutea* could potentially mitigate further weight gain. When the mice were switched from high caloric to a normo-caloric intake, it did not lead to statistically significant gain or loss of weight, in the presence or absence of the *F. lutea* ethyl acetate fraction. This indicates that the extract fraction in itself was not a weight loss agent. The failure of the mice to lose weight when switched from the high caloric to the normo-caloric diet was also not unexpected as other authors found that switching to a low caloric diet is required for a substantial weight loss [30, 32]. This was similar to findings of Veerapur et al. [33], who observed that *Ficus racemosa* extract did not affect body weight and food intake in high fat diet (HFD) fed Albino Wistar male rats. Also supplementation of feed with cyanidin 3-glucoside did not affect the body weight in either HFD and *db*/*db* male mice or significantly change food intake during the experimental period [34]. Hou et al. [35] found no significance body weight change between the group receiving a normal standard diet and high carbohydrate – high fat diet (HC-HF) as well as between the groups of the HC-HF diet and metformin administration.

Further evidence supporting the inability of the ethyl acetate fraction of *F. lutea* to stimulate weight loss can be seen with the failure of the extract to induce a change in either faecal weight or the extent of nutrient absorption in the mice per treatment group in comparison to their control group. This differed from other studies, where the faecal weights of animals fed polyphenol-rich diet were significantly higher than those on a control diet [36, 37]. These authors speculated that polyphenols adsorb cholesterol, bile acids and dietary lipid thereby increasing faecal excretion. The reason for the failure of the ethyl acetate fraction to induce similar changes is unclear, but it could be speculated that in contrast to other studies, the ethyl acetate fraction of *F. lutea* does not inhibit the activity of pancreatic lipase, a key enzyme in the digestion and absorption of fat [36, 37].

An important result of this study was the decrease in the AUC of the glucose concentration versus time profiles in those animals that were treated with both a change in diet and the plant extract. With all the treatment groups showing the same time to peak in blood glucose concentrations (Tmax), conventional pharmacological principles indicate that the induced changes were not due to a change in the rate of glucose absorption, but rather to a change in the total extent of absorption or an increased depletion rate of plasma concentrations subsequent to absorption. Based on the *in vitro* effect of the plant extract, both these effects are possible. However considering that the plant extract failed to induce a change in faecal weights, the more likely mechanism is an increase in the depletion of glucose within the plasma. Since the latter is linked to increased uptake within the cells, the most likely mechanism is either an increased insulin response or increased insulin secretion. From the *in vitro* work, we believe that the effect is due to the stimulation of insulin secretion and perhaps not inhibition of activities of the α-amylase and α-glucosidase.

## Conclusion

The polyphenol-rich ethyl acetate fraction of *F. lutea* extract was the most active fraction. In *in vitro* assays, ethyl acetate fraction, an intermediate polarity solvent, potently inhibited α-glucosidase activity, enhanced superior glucose uptake of muscle and liver cells, and stimulated insulin secretion. For the *in vivo* assays, although the ethyl acetate fraction of *F. lutea* extract exerted no statistical significant weight loss benefit in the treatment groups compared to their control, it significantly attenuated AUC profiles in the animals that were treated with both a change in diet and the plant extract. Future work will be to investigate the changes in plasma insulin concentrations following treatment with the ethyl acetate fraction of crude *F. lutea* acetone polyphenol extracts to determine the mechanisms of ameliorating hyperglycaemia.
